# A Multi-Controller Embedded Intelligent Crane System with Integrated Fire Safety for Light-Load Material Handling

**DOI:** 10.3390/s26103017

**Published:** 2026-05-11

**Authors:** Zhangwen Huang, Jiayang Song, Yuxiang Shi, Haichen Zhang, Chengyu Wang, Peijin Chen, Chunjiang Shuai

**Affiliations:** 1 Trine Engineering Institute, Shaanxi University of Technology, Hanzhong 723000, China; huangzhangwen@snut.edu.cn (Z.H.); songjiayang@snut.edu.cn (J.S.); shiyuxiang@snut.edu.cn (Y.S.); zhanghaichen@snut.edu.cn (H.Z.); chenpeijin@snut.edu.cn (P.C.); 2 Pei Yuan Academy, Shaanxi University of Technology, Hanzhong 723000, China; wangchenyu@snut.edu.cn; 3 Shaanxi Key Laboratory of Advanced Manufacturing and Health Management for Aviation Components, Shaanxi University of Technology, Hanzhong 723000, China

**Keywords:** intelligent crane system, Arduino, multi-sensor fusion, path planning, fire emergency response, embedded control, modular control

## Abstract

With the development of industrial intelligence, traditional material handling systems suffer from insufficient flexibility, low functional integration, and weak fire safety response. To solve these problems, this paper designs an Arduino-based multifunctional intelligent material handling crane system with integrated fire safety protection. The system adopts a modular multi-sensor fusion architecture, realizing environmental perception, automatic path planning, and dual fire safety protection (smoke alarm + automatic fire extinguishing). Experiments were carried out in a laboratory-controlled environment with the system in as the benchmark; the results show that the operation efficiency of object handling is improved by 29.6%. This prototype system provides an experimental reference for the intelligent and safe upgrading of small and medium-sized warehousing material handling equipment. All experiments were completed in a controlled laboratory environment.

## 1. Introduction

Thanks to increasing technological investment and industrial policy support over recent years, China has witnessed remarkable progress in industrial robotics [1]. Driven by fast-growing global trade, application scenarios for heavy-duty material handling robots have expanded from conventional factory workshops to complex operational environments, including ports and large-scale warehousing logistics centers. The latest statistical data indicates that as of July 2024, China boasted over 190,000 valid patents in robotics technology, accounting for 66.7% of the global total, demonstrating significant technological leadership. The Chinese industrial robotics market has been ranking No. 1 in size globally for 11 consecutive years, representing the nation’s technological accumulation and market potential in intelligent manufacturing.

Research on material handling cranes has achieved considerable maturity abroad. This means increasingly expanding applications, particularly in logistics, transportation and automated flexible production lines. Though existing systems are designed for autonomous handling tasks, their cargo volume and scale capacities are yet to be improved, failing to meet large-scale operational demands. To align with industry requirements, scholars have conducted more in-depth research on handling robots. For example, Canada’s Carpathian Robotics developed the OTTO heavy-load transport vehicle capable of bulk material handling and resolving repetitive material transport in production workshops for better operational efficiency [2]. Unimicron has launched a new generation of visual navigation AGVs, which are applied to the intelligent logistics hubs of automotive parts manufacturing plants by integrating stereo vision and LiDAR technology, solving the dynamic obstacle avoidance and path planning problems in complex industrial scenarios and improving the daily handling efficiency of a single machine [3].

Despite the relatively mature foreign crane technologies, China’s material handling robotics development is struggling with high costs. Although domestic universities and enterprises have made some autonomous technological breakthroughs—exemplified by Chang’an University’s intelligent mobile robots and Shenyang Xiyasi’s assembly line AGVs—persistent reliance on imported core components and excessive customized development come with surged production costs. This conflict between high R&D (research and development) investment and low-mass production directly disables the adoption of domestic robots in middle and small-sized warehousing scenarios [4,5].

Amid recent improvements, the logistics warehousing industry continues to confront multiple challenges: traditional manual handling proves low efficiency, high error rates, and frequent accidents, while a large number of warehouses suffer from slow-response fire systems and disorganized hazardous material management. Notable incidents include the fire at Korea’s Icheon logistics warehouse caused by unauthorized hot work [6] and the 2022 Amazon warehouse fire triggered by electrical failures [7]. Empirical studies confirm that such accidents not only cause severe casualties and property losses but also pose sustained negative impacts on supply chain systems. This necessitates the urgent need for intelligent transformation and enhanced safety management in warehousing logistics.

This paper proposes an Arduino-based intelligent material handling crane system that constructs an environmental perception, path planning, and a safety protection closed-loop through multi-sensor fusion technology, innovatively integrating flame/smoke detection modules with automatic fire suppression devices. The system aims to break dual bottlenecks in traditional solutions—insufficient grasping efficiency and emergency response capabilities—providing an integrated approach that combines high-efficiency operations with proactive safety measures for high-risk warehousing scenarios, thereby furthering industry-wide intelligent and safety-focused upgrades.

The main contributions of this paper are: (1) Proposing a modular Arduino-based intelligent handling crane integrating vision recognition and fire safety; (2) realizing closed-loop control of “perception-decision-execution” for handling and fire safety; and (3) verifying through experiments that the system improves handling efficiency by 29.6% and meets basic fire safety protection standards in laboratory environments.

## 2. Related Works

In recent years, intelligent material handling technology has been widely studied and applied in industrial settings. AGVs, robotic arms, and overhead cranes have become key equipment for automated logistics [8,9]. Many embedded control schemes based on Arduino have been proposed for educational and light-load robots, realizing basic motion control and sensor perception [10,11]. However, most of these systems focus only on handling functions and lack a safety protection design, especially fire detection and emergency response. With the improvement of warehouse safety requirements, fire detection and extinguishing technologies have been developed rapidly. Smoke sensors, flame sensors, and temperature sensors are widely used in fire monitoring systems [12,13,14]. Systems based on STM32 have shown advantages in real-time performance and power consumption for environmental monitoring [15,16]. Nevertheless, such fire safety systems usually operate independently and are rarely integrated with material handling equipment to form a unified intelligent platform. In terms of vision-based handling, visual recognition and positioning technologies represented by OpenCV and YOLO series algorithms have been gradually applied to robotic grasping [17]. Raspberry Pi, as a low-cost embedded computing platform, provides support for image acquisition and lightweight algorithm deployment [17]. However, existing solutions seldom combine visual handling, motion control, and fire safety protection into one compact system, resulting in a single function, high cost, or poor scalability, which cannot meet the application needs of small and medium-sized warehouses with limited cost and space. To address the above gaps, this paper designs a multifunctional intelligent crane system based on the collaborative work of Arduino, Raspberry Pi, and STM32. It integrates visual handling, path planning, and fire safety in one set of equipment, realizing high integration, low cost, and reliable safety performance.

## 3. Analysis of the Overall Design Scheme for the Multifunctional Logistics Handling System

**Figure 1:**
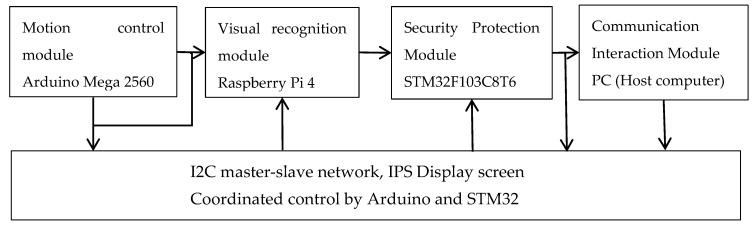
Overall system design block diagram.

This system primarily describes the design of a multifunctional intelligent material handling system based on the Arduino Mega 2560 control platform, consisting of four major functional modules: motion control, visual recognition, communication interaction, and safety protection [18]. The motion control module employs a robotic arm and a 42-step motor drive system to achieve precise grasping and transportation capabilities. Related research has validated the effectiveness of the PWM control strategy based on Arduino Mega 2560 for motor speed regulation and positioning control [19]. The visual recognition module constructs a position feedback network using multiple infrared sensors and employs an OpenCV-based modified YOLOv5 algorithm for multi-object recognition. The existing literature has successfully applied the modified YOLOv5 algorithm to multi-category object detection in robotic material handling scenarios [20]. The communication interaction module builds a master-slave network based on the I2C protocol, integrated with an IPS industrial display for real-time status monitoring. The safety protection module employs multi-sensor fusion technology, integrating smoke detectors and dual-band flame sensors to establish a tiered alarm mechanism. The complete system architecture is depicted in Figure 1. The Arduino controller is responsible for motion control, robotic arm actuation and execution mechanism control; the Raspberry Pi undertakes visual recognition, image acquisition, OpenCV-based image processing and path planning; the STM32 controller is used for multi-sensor data acquisition, fire safety monitoring, alarm signal output and fire extinguishing actuation control; the PC serves only as a host computer for system debugging, status monitoring and data display, and does not participate in the real-time embedded control process.

Overall System Design Block Diagram. The system takes Arduino Mega 2560 as the core, including four modules: motion control, visual recognition, communication interaction, and safety protection. The motion module drives the mechanical arm and stepping motor; the visual module completes object recognition based on OpenCV and improved YOLOv5; the communication module uses I2C protocol and IPS display for human–computer interaction; the safety protection module realizes smoke detection, flame detection and hierarchical alarm.

## 4. Functional Module Working Principle

This system adopts a modular design idea, and the core control, mechanical structure, kinematics, visual recognition, multi-sensor monitoring, fire extinguishing and smoke alarm modules are designed in turn. The overall logic follows “environmental perception → decision planning → action execution → safety feedback”.

### 4.1. Control Module

**Figure 2:**
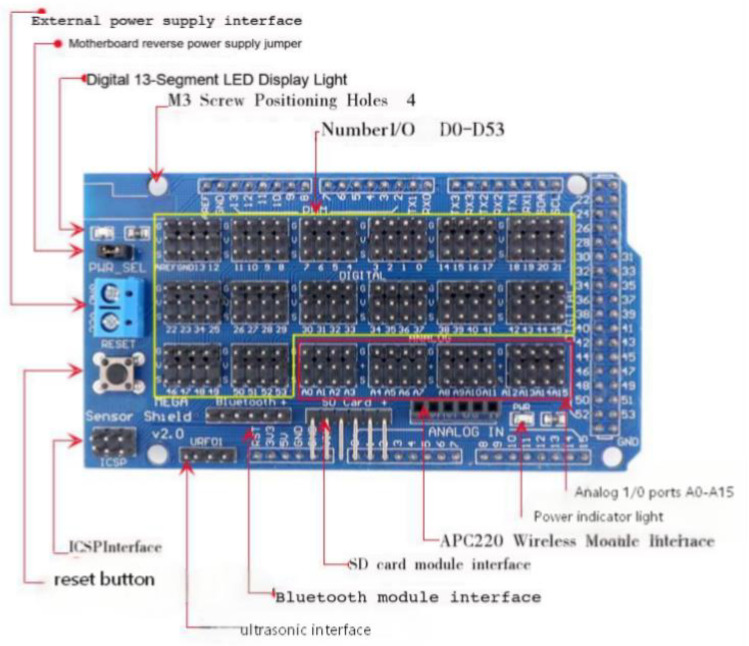
Arduino Mega 2560. Source: OpenMV, “OpenMV Cam M4 V1,” OpenMV Inc., 2026. Available online: https://openmv.io/products/openmv-cam-m4-v1 (accessed on 8 July 2025).

The core control unit of the system is the Arduino Mega 2560 microcontroller, which provides sufficient hardware resources for multi-sensor integration and motion control tasks, including 54 digital I/O pins, 16 analog inputs, and 4 hardware UART interfaces [20]. To simplify the wiring and connection of various peripheral devices, a dedicated Sensor Shield V2.0 expansion board is adopted, as shown in Figure 2. This shield is designed to plug directly onto the Arduino Mega 2560, expanding all the microcontroller’s digital (D0–D53) and analog (A0–A15) pins into convenient terminal blocks. It also integrates dedicated, pre-defined sockets for common modules such as Bluetooth, ultrasonic sensors, SD cards, and APC220 wireless communication modules. Additional features include a reset button, a power indicator light, and an external power supply interface, significantly improving the system’s expandability, maintainability, and ease of assembly.

### 4.2. Mechanical Structure Design

**Figure 3:**
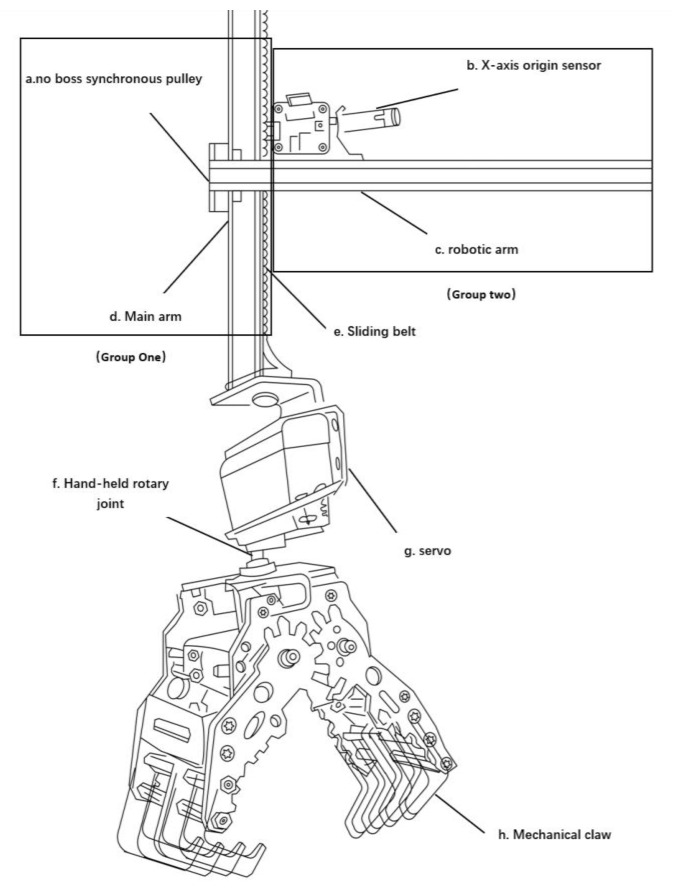
Mechanical Arm Structure Diagram.

The robotic arm of the material handling crane is divided into two primary components: the posture adjustment system and the grasping system, with specific construction details illustrated in Figure 3 [21].

The posture adjustment system consists of the handling crane arm, main arm, and non-flanged synchronous pulley. The first group comprises the crane’s main arm, sliding belt, and non-flanged synchronous pulley, and the second group includes the robotic arm and *X*-axis origin sensor. This vertical posture adjustment design ensures stability during material handling operations, helping maintain a consistent working position and orientation.

The grasping system employs a servo-driven mechanism incorporating a rotating wrist joint and a mechanical claw. When the front and rear arms move under horizontal and vertical slider guidance, the main arm simultaneously coordinates its movement [22].

### 4.3. Kinematic Modeling Module

**Figure 4:**
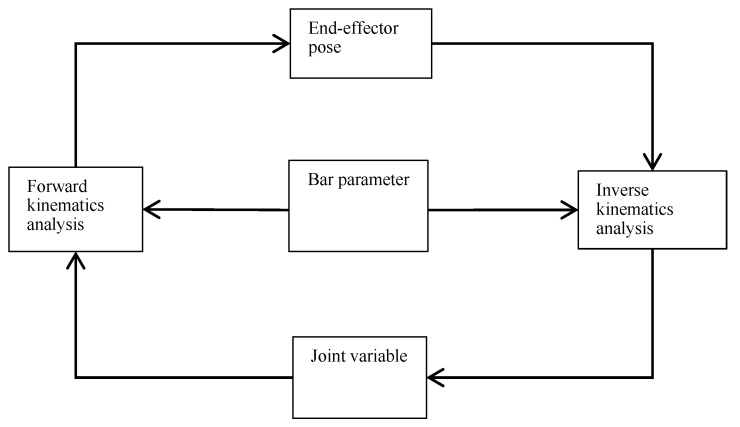
The forward and inverse kinematics of the crane.

Motion planning of the material handling crane constitutes the foundation of the control system, with recent research on industrial crane systems in three-dimensional space establishing bidirectional mapping between system inputs and outputs for efficient trajectory generation while explicitly considering kinematic constraints to ensure safe and feasible motions [23]. This correlation is schematically depicted in Figure 4. The framework encompasses two principal computational domains: forward kinematics (FK) and inverse kinematics (IK) [24].

**Figure 5:**
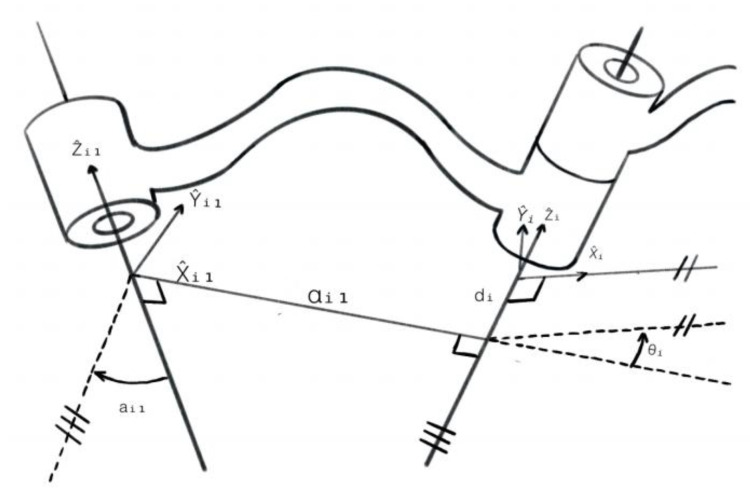
Schematic diagram of connecting rod parameters. Source: Craig, J. J. (2006) [25]. Introduction to robotics (Y. Chao, Trans.; 3rd ed.). China Machine Press. (Original work published 2005).

Model and number the manipulator using the D-H parameter method, and establish the mechanism coordinate system. When employing the standard D-H parameter method, the coordinate system of the linkage mechanism is set as “i + 1,” aligning it with hinge i in a straight line. In this approach, a modified D-H parameter method is adopted, utilizing a new coordinate system where the coordinate system (i + 1) aligns with hinge I-1, thereby altering the transfer matrix between links. Based on this, a D-H parameter-based loading crane model is proposed as shown in Figure 5 [24]. Determine the common perpendicular line or intersection point between hinge axis i and the next hinge axis, and designate the Intersection point between hinge axis i or the common perpendicular axis and hinge axis i as the origin of the connecting coordinate system (i). Set the direction of connecting axis i as $Z_{i}$. If hinge axis i intersects with the next axis, axis $X_{i}$ is defined as perpendicular to the plane formed by the two axes. The *Y*-axis is determined according to the right-hand rule [25].

After establishing the mechanism coordinate system, the first step involves determining four kinematic parameters for each member, as detailed in the subsequent section. In this model, $a_{i}$ and $\alpha_{i}$ characterize the intrinsic geometric properties of individual links, and the remaining two parameters specify the spatial relationships between adjacent links. These parameters are defined as follows:

$A_{i}$: The translational displacement along the $X_{i}$ axis between the $Z_{i}$ and Z{i+1} axes, representing the link length.

$a_{i}$: The rotational displacement about the $X_{i}$ axis from $Z_{i}$ to Z{i+1} describing the twist angle between links.

$d_{i}$: The translational displacement along the $X_{i}$ axis from X{i−1} to *X_i_*, quantifying the link offset.

$\theta_{i}$: The rotational displacement about the $Z_{i}$ axis from X{i−1} to *X_i_*, defining the joint angle.

### 4.4. Recognition Module

**Figure 6:**
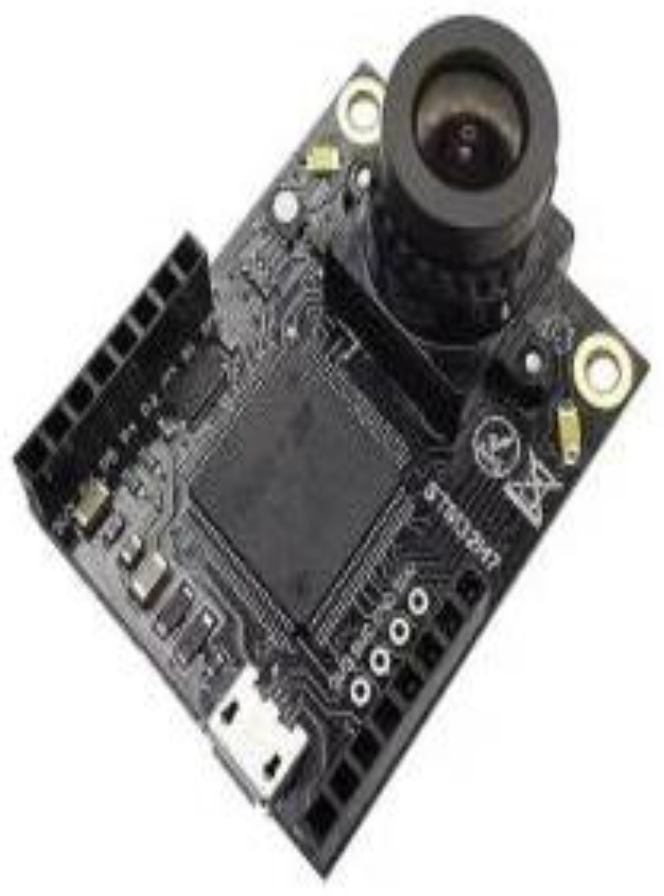
The OpenMV vision module equipped with an OV5640 camera [27]. (accessed on 8 July 2025).

Its visual recognition module uses the OpenMV Cam H7 Plus (Figure 6 [26]) (Guangzhou Singtown Information Technology Co., Ltd., Guangzhou, China) as the embedded vision core. This module integrates the OmniVision OV5640 5-megapixel CMOS sensor (supporting 1080P/30fps video capture with a 3.6 mm focal length lens) for stable image acquisition in industrial warehouse environments, and works in conjunction with a Raspberry Pi 4 Model B (64-bit 1.5 GHz quad-core, 4 GB RAM).

**Figure 7:**
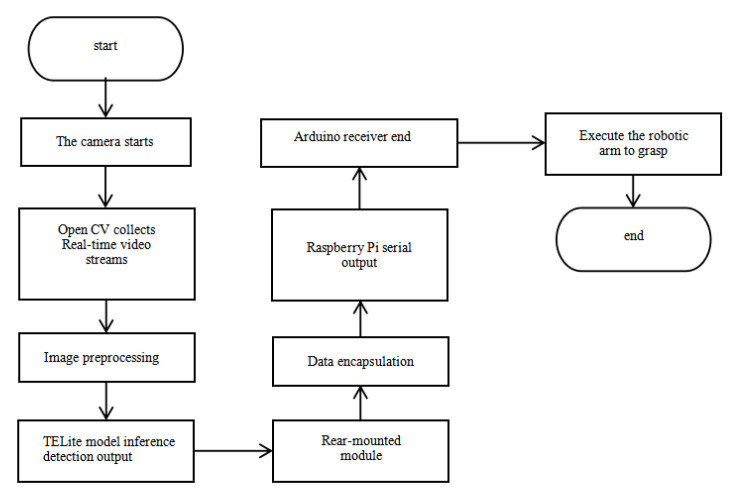
Working principle diagram.

The detection data is packaged using the UART protocol prior to transmission. The post-processing module computes spatial transformations to obtain Cartesian coordinates compatible with the robotic arm. The Arduino controller drives servo motors through asynchronous serial communication to execute grasping operations, as seen in Figure 7.

### 4.5. Intelligent Monitoring Feedback Module with Multi-Sensor Fusion

The system adopts a multi-controller collaborative architecture, in which Arduino Mega 2560 serves as the main controller for motion execution and overall scheduling, while STM32F103C8T6 acts as a dedicated co-processor for multi-sensor fusion and intelligent monitoring feedback. This hierarchical design ensures real-time performance and reduces the computational load of the main control unit.

The multi-sensor fusion monitoring feedback module mainly consists of the STM32F103C8T6 core, LCD1602 display, HC-05 Bluetooth communication module, DS18B20 temperature sensor (−55 °C to +125 °C), MQ-135 smoke sensor, flame detection module, relay-driven water pump, ventilation fan circuit, and audible-visual alarm device. The STM32F103C8T6 is selected for its superior accuracy and power efficiency in environmental monitoring compared with traditional 51 MCUs and general Arduino platforms. Sensors, including DS18B20 and MQ-135, are adopted to achieve reliable real-time sampling of temperature and smoke concentration [28]. The HC-05 Bluetooth module supports wireless data interaction between the monitoring module and the upper computer, with stable and low-power communication performance verified in similar environmental monitoring systems [29].

**Figure 8:**
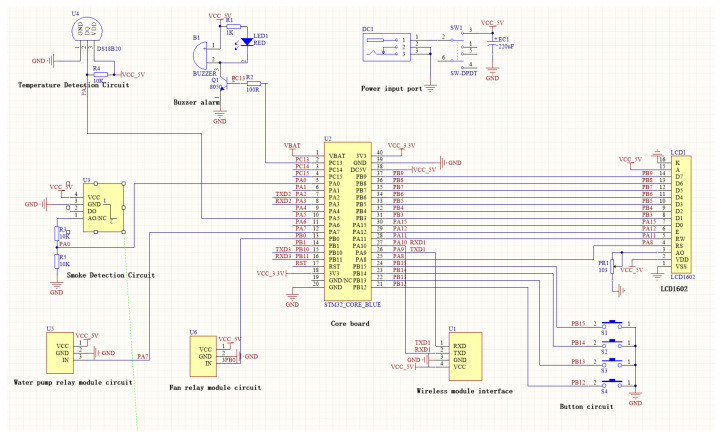
Schematic diagram of an intelligent monitoring feedback system with multi-sensor fusion.

The STM32 co-processor collects and fuses multi-dimensional environmental data, then transmits alarm and status signals to the Arduino main controller through serial communication. The main controller then coordinates the actions of mechanical arms, actuators, and fire safety components, forming a complete closed loop of “perception–decision–execution”. The complete multi-sensor monitoring system circuit is illustrated in Figure 8.

### 4.6. Fire Extinguishing Module

The fire extinguishing module of this system comprises a far-infrared flame sensor and a relay-controlled water pump assembly. The flame sensor converts infrared radiation intensity into analog signals through photoelectric conversion, with subsequent analog-to-digital conversion generating digital detection values inversely proportional to flame intensity. Research on ultraviolet flame detection systems has demonstrated effective conversion of photogenerated analog current signals to digital voltage signals through trans-impedance amplification and analog-to-digital conversion, providing a methodological foundation for flame signal processing [30]. Using a DC12V micro-pump changes the system spray intensity to 15 L/min/m^2^ (1.5 L/min ÷ 0.1 m^2^), which is far beyond the minimum spray intensity required for local application systems. Class A fires require a minimum spray intensity of 5 L/min/m^2^ [31]. The execution unit utilizes a transistor current amplification circuit to drive a 5 V relay, which activates a centrifugal water pump to form a spray fire suppression system. The system sprinkler intensity of 15 L/min-m^2^ far exceeds the minimum sprinkler intensity for localized applications: ≥5 L/min-m^2^ for Class A fires [32]. When smoke or gas is detected, the system activates the exhaust fans, and when a fire is detected, the water pumps start running [33].

#### Fire Extinguishing Execution Flow

(1) Audible and visual alarm activation;

(2) Water pump acceleration;

(3) Targeted water spray.

**Figure 9:**
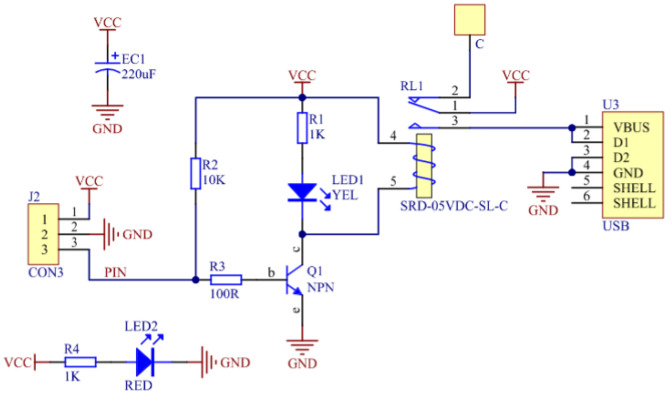
Relay module circuit diagram.

This implements an intelligent closed-loop fire extinguishing mechanism following the “perception-decision-execution” principle [34]. The corresponding relay circuit diagram is presented in Figure 9.

### 4.7. Smoke Alarm Module

When the smoke sensor collects real-time data, it transmits analog signals to the AD module for processing. After initialization, the system performs analog-to-digital conversion to obtain current smoke concentration values. The acquired data is then read and converted into digital quantities, which are subsequently transformed into corresponding voltage values for sequential output. The data processing description is as follows:

The formula for AD conversion voltage calculation:


(1) $$\begin{array}{c} V_{out}=\frac{AD}{2^{n}}\times V_{ref} \end{array}$$

The calculation formula for smoke concentration:

(2) $$ppm=(V_{out0}-V_{out})\times k$$

where:

**V**_**out**_ is the actual output voltage (**V**); **AD** is the digital value after **AD** conversion;

**n = 10** is the **AD** conversion resolution of the Arduino;

**V_ref_ = 5 V** is the system reference voltage;

**V_out0_** is the real–time measured voltage (**V**);

**k** is the calibration coefficien to **f** the MQ–135 sensor;

ppm is the smoke concentration value.

## 5. Object Handling Experiment and Analysis

### 5.1. Construction of the Experimental Platform

The implementation of a vision-based object-handling platform demands comprehensive integration of hardware and software. The hardware architecture consolidates all device controls to the PC through standardized interfaces, while the software framework incorporates image processing algorithms, camera operations, and crane control modules into a unified programming environment via driver configurations. This ensures real-time system-wide operation on the PC [35]. The experimental platform specifications are detailed below:

(1) Hardware Components

The main hardware components include:

Handling crane system;

high-performance desktop computer;

OpenCV-compatible camera lens assembly;

flush-mount synchronous pulley mechanism;

precision servo motor;

42-axis synchronous stepper motor;

programmable mechanical gripper unit;

dedicated gripper controller.

(2) Software Support

The software and platforms used in the system include:

MATLAB R2023b computational environment;

AutoCAD 2021 mechanical design suite;

OpenCV computer vision library;

VS2008 integrated development environment;

Raspberry Pi embedded platform;

Keil μ5 embedded development toolkit.

#### Test Protocol

All experiments were carried out in a controlled laboratory environment with stable illumination and no external interference, ensuring reliable and repeatable test data. The test objects included wooden blocks, water bottles, standard cubes, and spheres, with a weight range of 0–0.5 kg to cover typical light-load handling conditions. The handling distance was set to 1.2 m, and the motion speed was carefully tuned to balance stability, positioning accuracy, and operational efficiency. Each test was repeated 4 times, and the average values were adopted as the final performance indicators.

### 5.2. Route Planning

**Figure 10:**
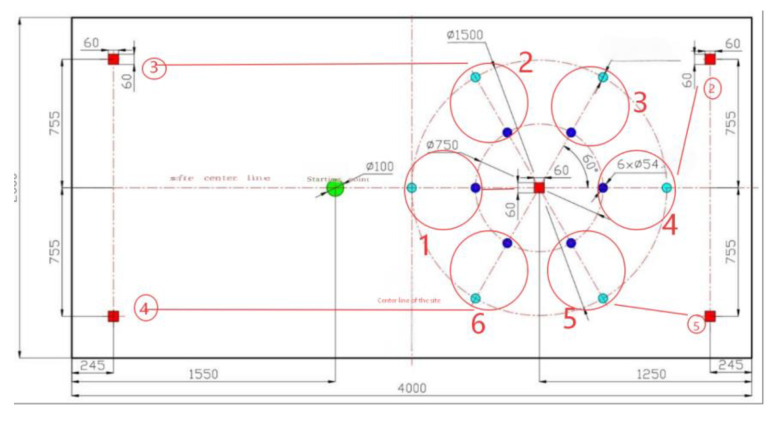
Experimental site planning.

The device is powered on, the lifting axis and the slide axis. The rotating axis returns to the origin and initializes to the initial position. The manipulator resets to the position of the first outer ring to be identified, see Figure 10. The motion program is shown below.

Switch (FlowControl State) {
//State 3: Move to the first station outer circle recognition position
Case 3:

1. Send stop command to all motion drivers
2. *X*-axis (Rotary Table) moves to the first detection position
3. *Y*-axis (Lifting Device) moves to visual recognition height
4. *Z*-axis (Crane Slide) moves to the first detection position

5. Switch state to 4 (next detection step)

//State 7: Weight detected, execute grasping motion
Case 7: 1. *Z*-axis moves to outer ring grasping position

2. *Y*-axis adjusts to grasping height
3. Activate manipulator: rotate servo + gripper clamp weight
4. *Y*-axis lifts weight to avoid collision
5. *Z*-axis moves to 10 cm wooden pile release position

6. Switch state to 15 (weight release step)

//State 20: No weight detected, move to station 4 inner circle recognition
Case 20: 1. *Z*-axis moves to station 4 detection position

2. Reset gripper servo to safety release state
3. *Y*-axis returns to weight recognition height
4. Switch state to 21 (inner circle weight detection) }

### 5.3. Intelligent Handling Crane Object Transportation Experiment

#### 5.3.1. Control Process for Object Movement

The vision-based object-handling system utilizes real-time visual feedback to determine target pose parameters, facilitating precise transportation of variably oriented objects through sequential handling operations.

The operational sequence comprises three phases:

First, software initialization is performed for the handling crane, camera, Raspberry Pi, and OpenCV in the system. The software triggers the industrial camera to capture images of the object and acquires the object’s pose parameters through image processing. The processed object position and pose parameters are then converted into the handling crane’s coordinate system parameters.

**Figure 11:**
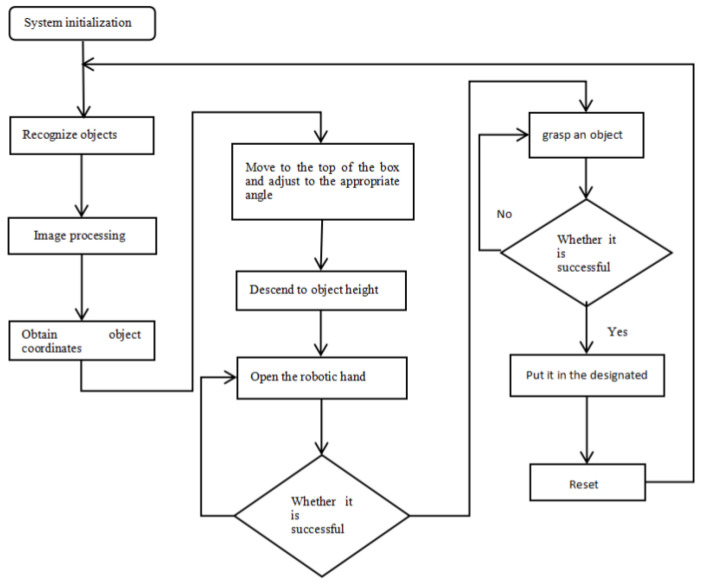
Operating process chart.

Second, the actual object pose is applied to automatically plan the motion path of the handling crane. The manipulator’s end-effector is moved to a position above the object, with the gripper rotated to match the object’s orientation angle. The handling crane is then controlled to descend to the object’s position for grasping and transports the object to the target location through the crane’s operating trajectory. Finally, it returns to the initial position to complete the cycle [35]. The operation flowchart is shown in Figure 11.

#### 5.3.2. Object Transportation Experiment

**Figure 12:**
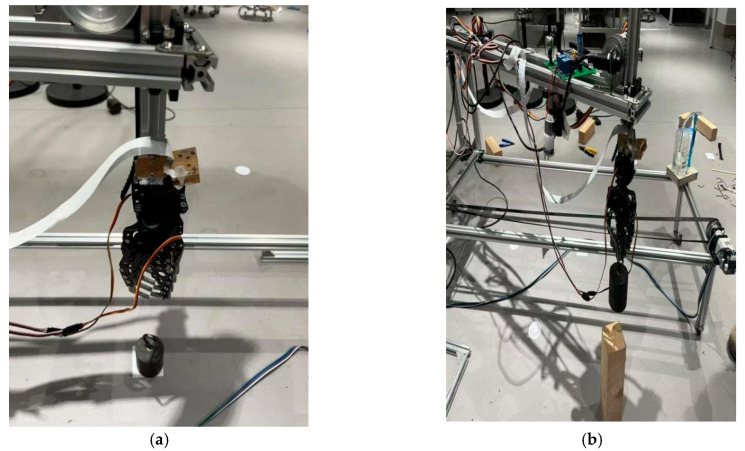

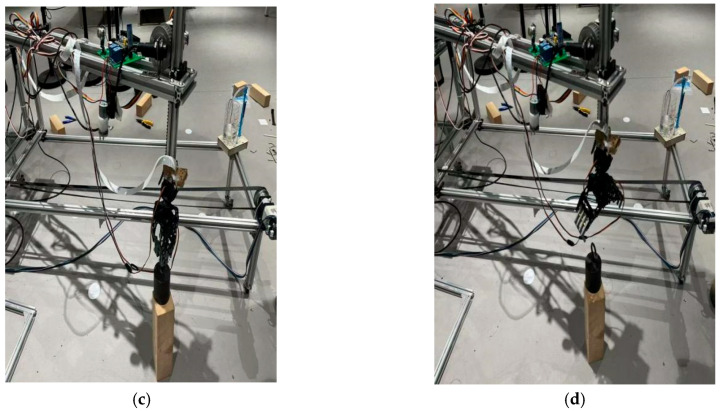
The experimental process of object transportation. (**a**) The mechanical claw moves to the position directly above the object. (**b**) The robotic claw grasps the object and moves it above the designated position. (**c**) The robotic claw grasps the object and moves it above the designated position. (**d**) The mechanical opens the claw after completing the grab.

The first group of experiments: The crane’s motion trajectory is automatically planned after acquiring the object’s position and orientation coordinates in the crane coordinate system through image processing. The robotic arm initially locates the target object via visual recognition and then moves to the position directly above the object while adjusting the gripper’s orientation to ensure secure grasping. Upon successful closure and gripping of the object, the robotic arm steadily elevates and transports it along the planned path to the position directly above the target location. The arm then gradually descends to precisely position the object at the designated spot for stable placement. After completing the placement task, the gripper automatically opens to release the object and returns to the standby mode for subsequent operations. Figure 12 demonstrates the complete object transportation process.

**Figure 13:**
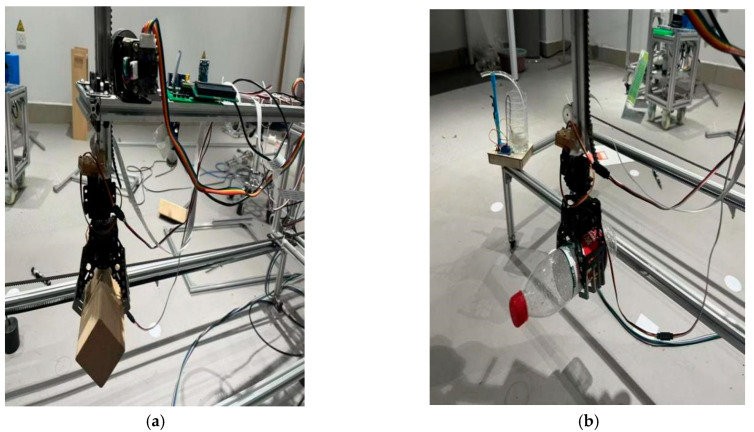

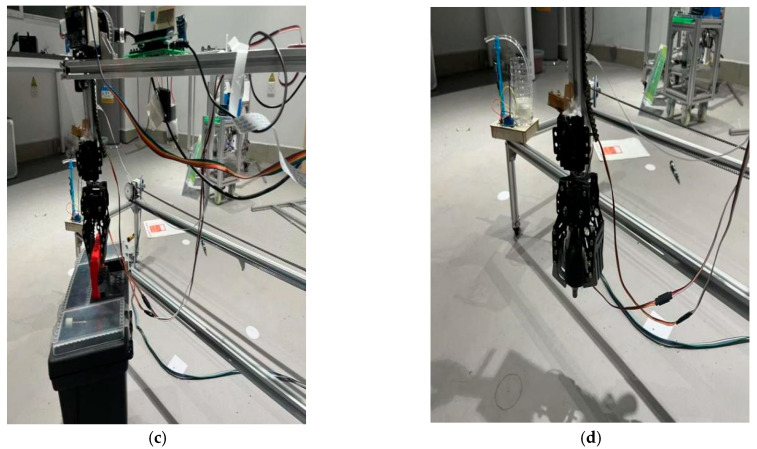
Second experiment process chart. (**a**) Claw grabs the block of wood; (**b**) claw grabs a bottle of water; (**c**) claw grabs the object; and (**d**) claw grabs the ball.

The second group of experiments: Identifying different treatments to grasp objects of varying shapes. Figure 13 illustrates the multi-shape object grasping procedure. Integration of both experimental groups confirms the following:

The material handling crane achieves programmed path accuracy for object positioning. Through controlled claw actuation, the system executes continuous, precise grasping/placement operations while demonstrating object recognition capability with grasping implementation. These findings validate the feasibility of the Arduino-based intelligent crane system for automated transportation of diverse objects.

#### 5.3.3. Experimental Results and Analysis of Object Handling

The results of multiple field tests with comparative analysis against Reference [36] indicate Table 1:

(1) An average grasping time of 19 s for the material handling crane;

(2) A mean object transportation time of 150.5 s;

(3) An overall 29.6% work efficiency improvement.

Through multiple field tests and comparative experiments with the design proposed in Reference [36], the results demonstrate that the optimized crane system delivers an average grasping time of 19 s and an average moving time of 150.5 s, representing an overall efficiency improvement of 29.6%. These findings validate the effectiveness of the proposed improvements, significantly reducing the operational cycle and providing a reliable basis for performance optimization of cranes in engineering practice. Future research could further investigate the system’s stability and adaptability. The average grasping time is shortened from 40 s to 19 s, and the average handling time is shortened from 200.8 s to 150.5 s. The efficiency improvement comes from optimized path planning, PWM motor control and visual positioning acceleration, which effectively shortens the idle stroke and adjustment time under varying working conditions to expand its applicability.

## 6. Fire Extinguishing Detection Experiment Results

**Figure 14:**
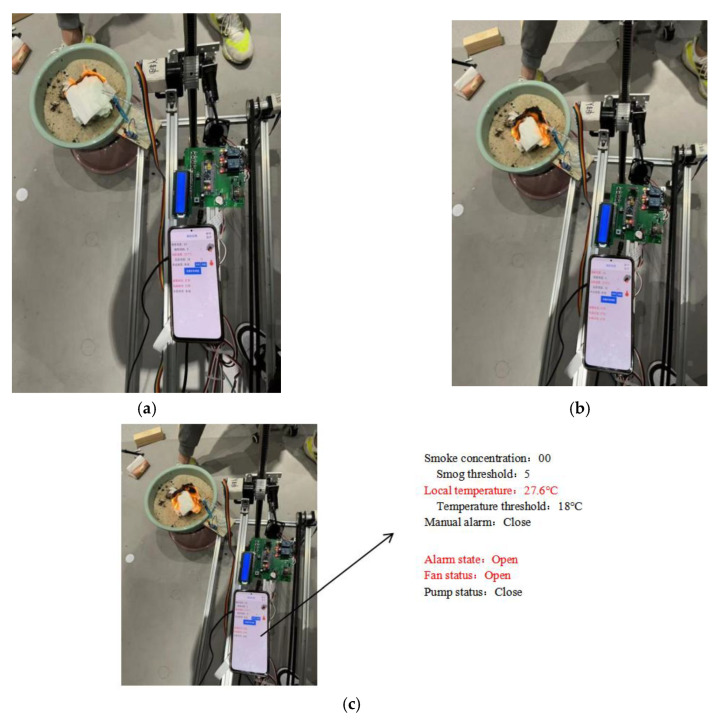
Intelligent detection experiment chart. (**a**) The alarm is activated when the temperature reaches 18 °C. (**b**) The water starts to pump to extinguish the fire. (**c**) When the flame is extinguished, the water starts pumping.

The test process is shown in Figure 14a–c. When a fire occurs and the temperature reaches the set value, the alarm and ventilation fan functions are triggered. When flames are detected, the water pump activates for fire extinguishing, while the ventilation fan starts when the smoke concentration reaches a certain level. The test results are shown in Table 2, Table 3 and Table 4.

Multiple experiments have verified that all functional modules of the system can make accurate responses according to preset thresholds:

(1) The alarm module can promptly trigger audiovisual alarm signals;

(2) The fan control unit can automatically activate based on temperature changes;

(3) The temperature detection system operates normally.

The fire extinguishing system demonstrates good operational stability in high-temperature environments (≥200 °C), capable of reducing the ambient temperature to below the safety threshold of 60 °C within (30 ± 5) seconds, extinguishing fire at the critical point where the material’s heat release rate approaches zero [26].

The system fully meets fire safety standard requirements. Experimental data indicates that all performance metrics of the system satisfy design specifications, demonstrating reliable fire prevention and control capabilities.

Supplementary explanation:

(1) The fire extinguishing experiment adopts laboratory-simulated fire sources and manual calibration data; no public fire dataset is used, and all data are collected by the system’s own sensors.
(2) All fire safety experiments are simulated tests in a controlled laboratory environment, not real industrial fire scenarios.
(3) The system is a small prototype, with a rated load of 0.5 kg; it is suitable for small and medium-sized warehouses and light-load handling scenarios. For large industrial cranes, it is necessary to upgrade the drive motor, load structure and communication mode.

## 7. Conclusions

This paper designs an Arduino-embedded multifunctional material handling crane with integrated fire safety. The system integrates visual recognition, precise handling and fire safety protection to form a closed-loop of “perception-decision-execution”. Experimental results show that: (1) Compared with the benchmark system [36], the operation efficiency is improved by 29.6%; (2) the fire safety module can complete alarm and fire extinguishing within (30 ± 5) s in the simulation environment; and (3) the modular design improves the system’s scalability and maintainability.

The system is a feasible prototype for light-load intelligent handling in small and medium warehouses. However, the current prototype still has limitations in load capacity, environmental adaptability and algorithm robustness for complex industrial scenarios. In addition, the current tests are limited by a few repetitions (n = 4) and a single experimental scenario.

Future work will focus on the following aspects: (1) Integrate dynamic event-triggered mechanisms and sensor fault-tolerant control strategies to improve stability in complex warehouse environments; (2) upgrade the hardware platform to enhance load capacity and motion stability; (3) introduce 5G communication to realize remote monitoring and cloud management; and (4) optimize the fire detection algorithm and expand the protection scope to multi-type fires.

These improvements will further promote the practical application of the system in intelligent and safe logistics warehousing.

## Figures and Tables

**Table 1:** Comparison of crane performance tests with literature [36].

Test Times	Grasping Time/s		Moving Time/s		Total Time/s	
	This Thesis	Literature [36]	This Thesis	Literature [36]	This Thesis	Literature [36]
1	20	41	150	198	170	239
2	18	42	149	203	167	245
3	21	40	152	199	173	239
4	17	39	151	198	168	237
Average time	19	40	150.5	200.8	169.5	240.8

**Table 2:** Temperature detection.

Setpoint Temperature (°C)	Test Value Temperature (°C)	Whether to Call the Police
18	16.1	No
18	17.6	No
18	26.2	Yes
18	27.6	Yes

**Table 3:** Smoke concentration detection.

Concentration Setpoint (ppm)	Concentration Test Value (ppm)	Fan Status
10	8	Close
10	10	Open
10	12	Open
10	15	Open

**Table 4:** Fire extinguishing function test.

Test Times	Highest Temperature (°C)	Time to Reduce to 60 °C (s)
The first time	314	28
The second time	306	25
The third time	320	30
The fourth time	338	36

## Data Availability

The datasets used and/or analyzed during the current study are available from the corresponding author on reasonable request.
